# Supplementary material for: Generation and Transcriptome Profiling of Slr1-d7 and Slr1-d8 Mutant Lines with a New Semi-Dominant Dwarf Allele of SLR1 Using the CRISPR/Cas9 System in Rice
Source: Int J Mol Sci. 2020 Jul 31;21(15):5492. doi: 10.3390/ijms21155492 (PMC7432230; doi:10.3390/ijms21155492)
Supplement: Supplementary file 1 [file ijms-21-05492-s001.zip › Supplementary Figures S1-S7 the final.docx]

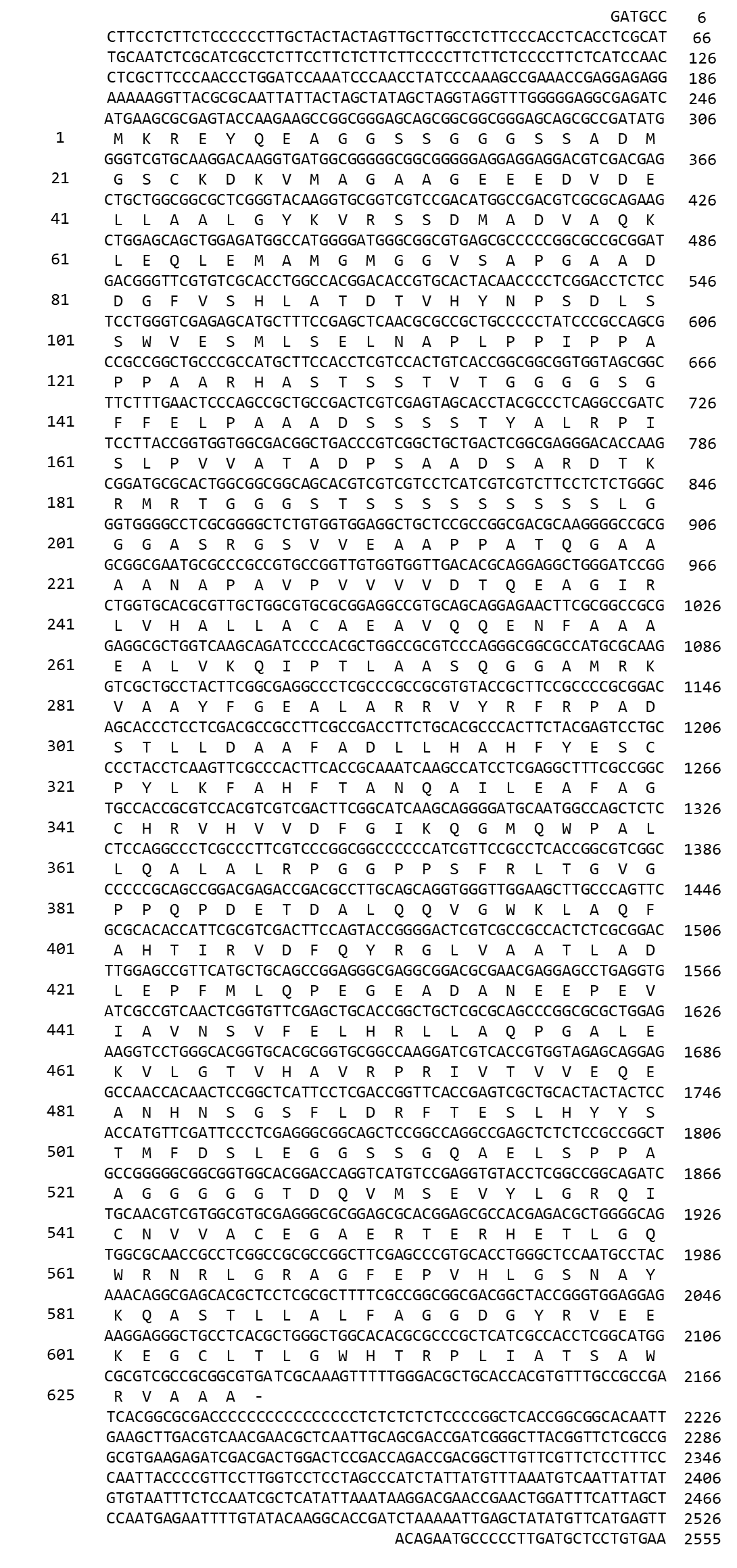


Supplementary Figure S1. Nucleotide and amino acid sequences of *SLR1* gene in rice.


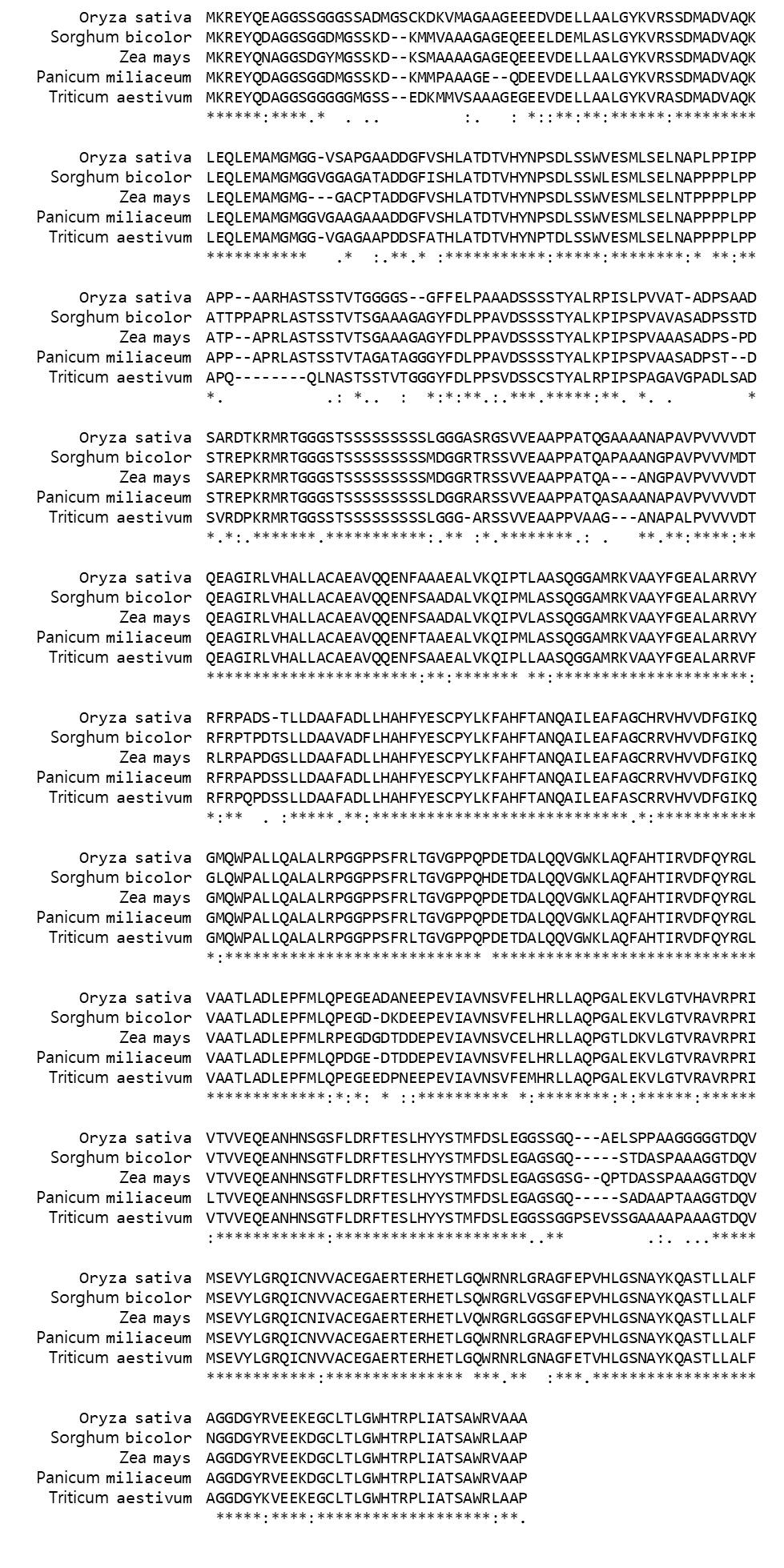


Supplementary Figure S2. Amino acid sequences alignment of the coding region of *SLR1* gene from *Oryza sativa*, *Sorghum bicolor, Zea mays, Panicum miliaceum* and *Triticum aestivum*.


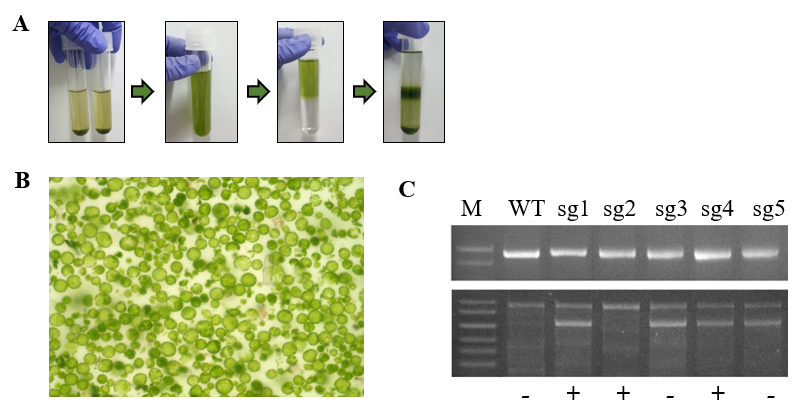


Supplementary Figure S3. Confirmation of the efficiency of sgRNA using T7-endonuclease I enzyme. (A) Protoplasts isolation from 10-days-old rice leaves. (B) Subjected to microscopy. (C) T7E1 assay for genome editing efficiency of *OsSLR1* sgRNAs by CRISPR/Cas9 system. PCR products were treated with T7E1 enzyme, and imaged negative (−) and edited pools (+) of the target sites.


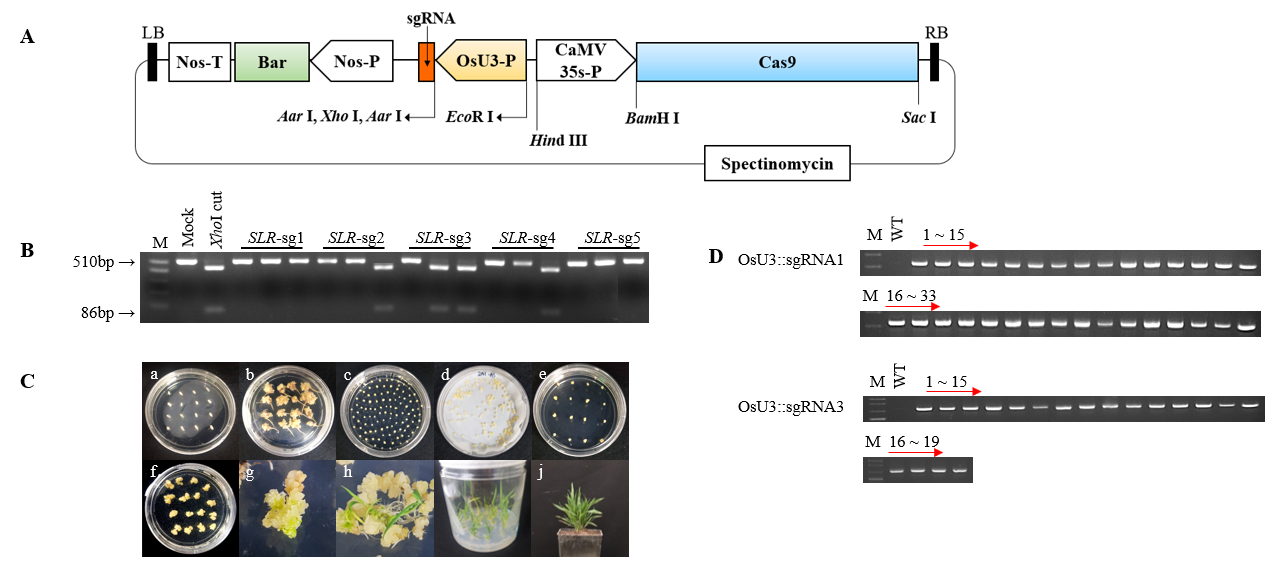


Supplementary Figure S4. CRISPR/Cas9 binary vector construction and rice transformation (A) Ti-plasmid vector construction for gene editing of *OsSLR1*. (B) Gel electrophoresis of PCR products for sequencing analysis from pBOsC:sgRNA vctor. WT, Mock and *Xho*I cut were used as controls. Lane *SLR*-sg1 to *SLR*-sg5 confirm the introduction of sgRNA into each vector. (C) Steps for generation of transgenic plants using *Agrobacterium* methods. (D) Confirmation of transgenic plants through PCR analysis.


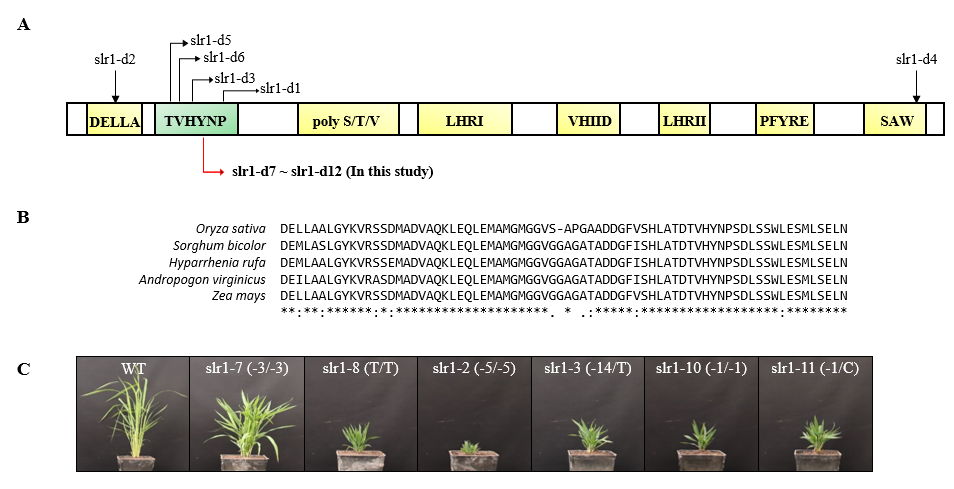


Supplementary Figure S5. Confirm of mutant phenotype and conserved domain region of DELLA protein. (A) Structure of the *SLR1* gene motif. (B) Comparison of the deduced amino acid sequences of DELLA proteins. Amino acid sequences alignment of DELLA proteins from *Oryza sativa, Sorghum bicolor, Hyparrhenia rufa, Andropogon virginicus* and *Zea mays*. The arrow indicated mutation sites of dwarf mutants. (C) Phenotypes of WT and edited homo mutant lines.


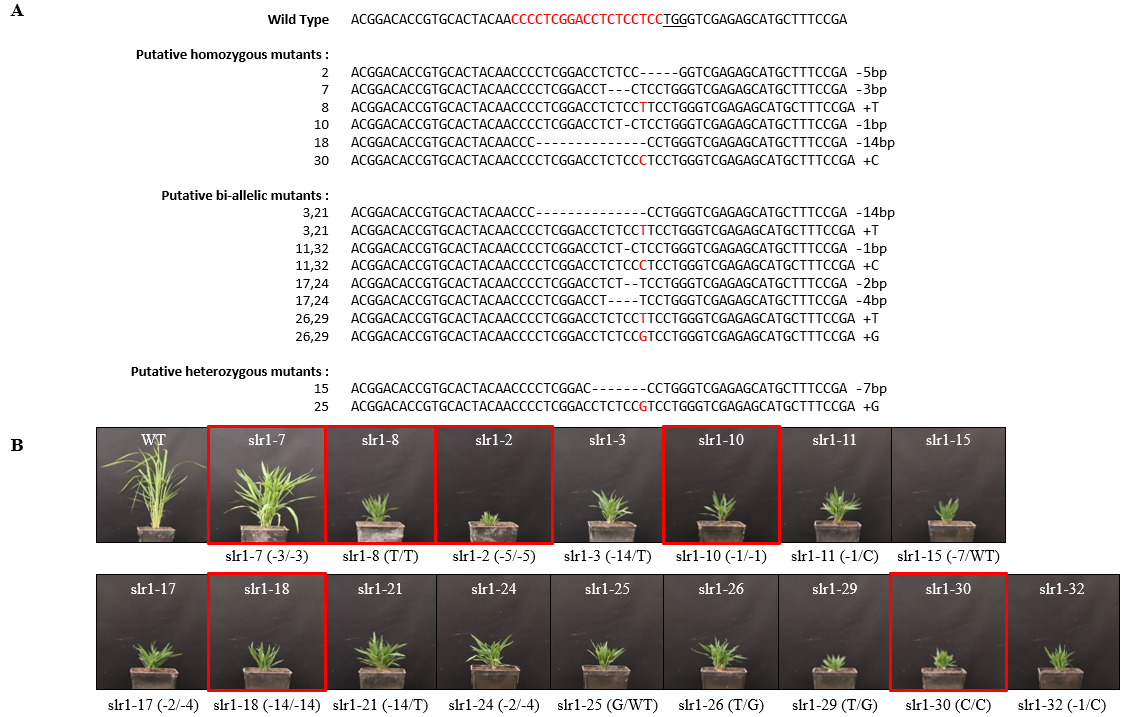


Supplementary Figure S6. CRISPR/Cas9-induced mutations in the *OsSLR1* gene and phenotype of edited plants. (A) The mutant *OsSLR1* genotypes of representative T0 plants are identified by DNA sequencing and alignment. Deletions and insertions are indicated by dashes and red letters, respectively. The numbers on the right side show the sizes of the indels, with “−” and “+” indicating deletion and insertion of the nucleotides involved, respectively. The letters after the numbers represent different bases of the same length. (B) Plant morphologies of sixteen edited mutant lines and WT plants were cultivated in the greenhouse for 2 months.


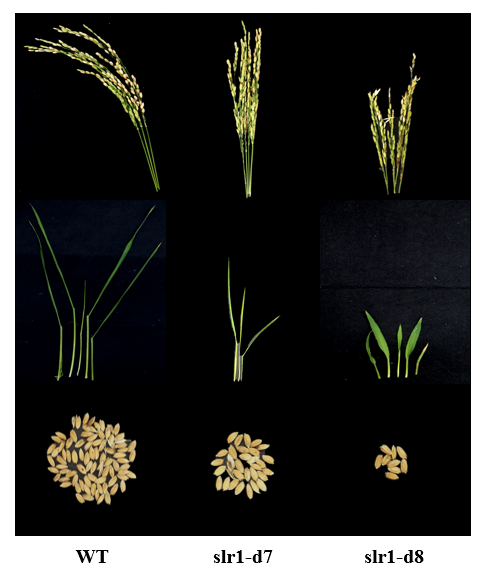


Supplementary Figure S7. Appearance of panicle, leaves and grains after harvesting slr1-d7 and slr1-d8 lines compare to WT
