# Supplementary material for: Generation and Transcriptome Profiling of Slr1-d7 and Slr1-d8 Mutant Lines with a New Semi-Dominant Dwarf Allele of SLR1 Using the CRISPR/Cas9 System in Rice
Source: Int J Mol Sci. 2020 Jul 31;21(15):5492. doi: 10.3390/ijms21155492 (PMC7432230; doi:10.3390/ijms21155492)
Supplement: Supplementary file 1 [file ijms-21-05492-s001.zip › Supplementary Table S1-S2.docx]

Supplementary Table S1. Design of sgRNAs for CRISPR genome editing on *OsSRL1* gene in rice.

| sgRNA | Target sequences (5' to 3') | Direction | GC Contents  (%, w/o PAM) | Out-of-frame Score | Mismatches | | | |
| --- | --- | --- | --- | --- | --- | --- | --- | --- |
|  |  |  |  |  | 0 | 1 | 2 | 3 |
| SLR1-sg1 | ACCCCTCGGACCTCTCCTCCTGG | + | 70.0 | 49.8 | [1](javascript:%20void(0);) | 0 | 0 | 0 |
| SLR1-sg2 | TGGACGAGGTGGAAGCATGGCGG | - | 60.0 | [72.3](javascript:%20void(0);) | 1 | 0 | 0 | 0 |
| SLR1-sg3 | TGGACGAGGTGGAAGCATGGCGG | - | 60.0 | 72.3 | 1 | 0 | 0 | 0 |
| SLR1-sg4 | TGGTTGACACGCAGGAGGCTGGG | + | 60.0 | 67.0 | 1 | 0 | 0 | 0 |
| SLR1-sg5 | GCATCAAGCAGGGGATGCAATGG | + | 55.0 | 68.8 | 1 | 0 | 0 | 0 |

Supplementary Table S2. Mutation rate and edited plant types for *OsSLR1* gene using CRISPR/Cas9 system.

| Targeted region | No. of Transgenic plants | No. of no edited plants | No. of edited plants | Mutation  Rate  (%) | Genotype | | |
| --- | --- | --- | --- | --- | --- | --- | --- |
|  |  |  |  |  | Homo | Hetero | Bi-allelic |
| SLR1-sg1 | 31 | 15 | 16 | 48.4 | 6/16 | 2/16 | 8/16 |
| SLR1-sg3 | 19 | 19 | 0 | 0.0 | - | - | - |
