# Supplementary figures and images for: Generation and Transcriptome Profiling of Slr1-d7 and Slr1-d8 Mutant Lines with a New Semi-Dominant Dwarf Allele of SLR1 Using the CRISPR/Cas9 System in Rice
Source: Int J Mol Sci. 2020 Jul 31;21(15):5492. doi: 10.3390/ijms21155492 (PMC7432230; doi:10.3390/ijms21155492)

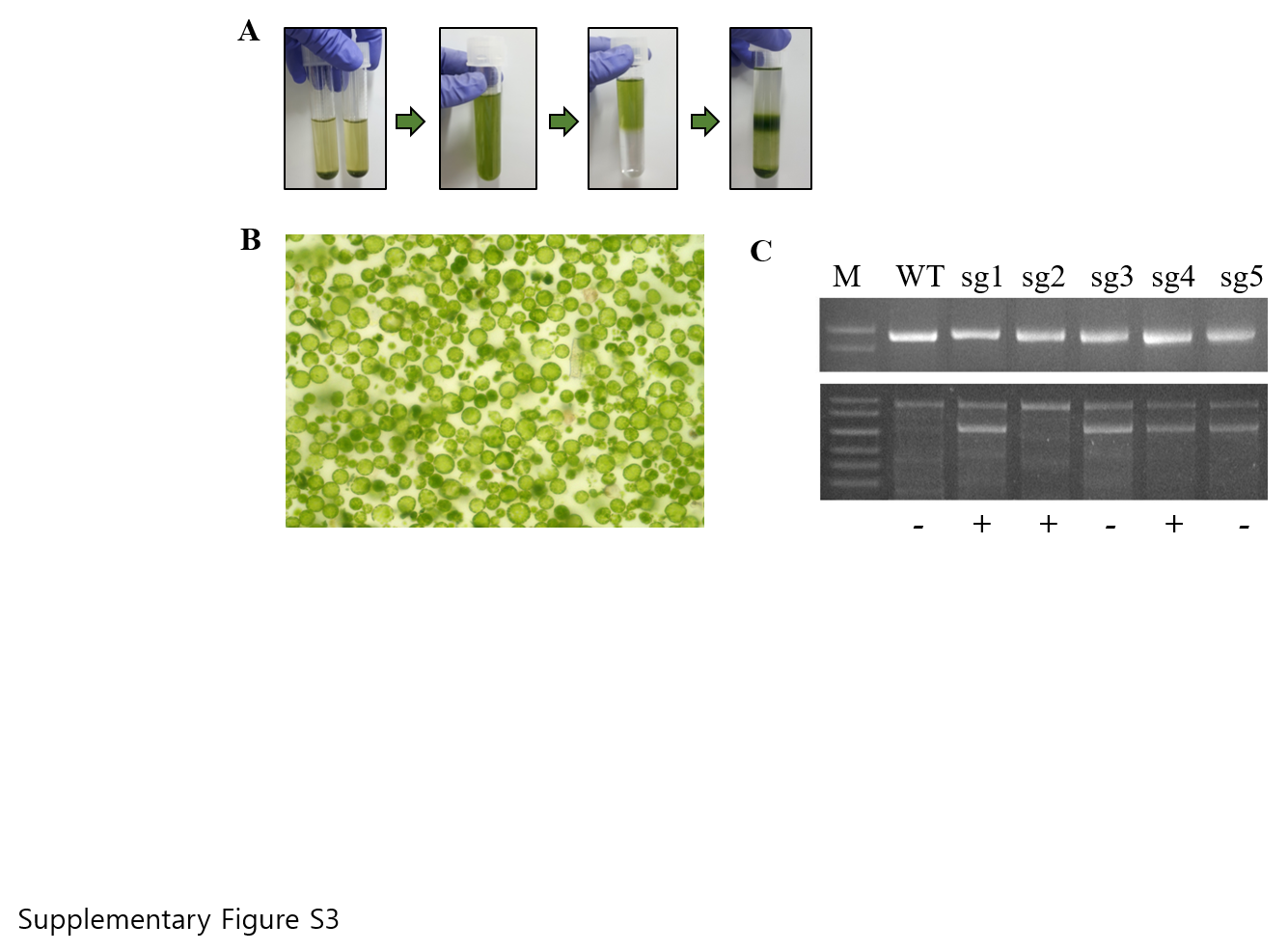

Supplement: Supplementary file 1 [file ijms-21-05492-s001.zip › Supplementary Figure S3.tif]

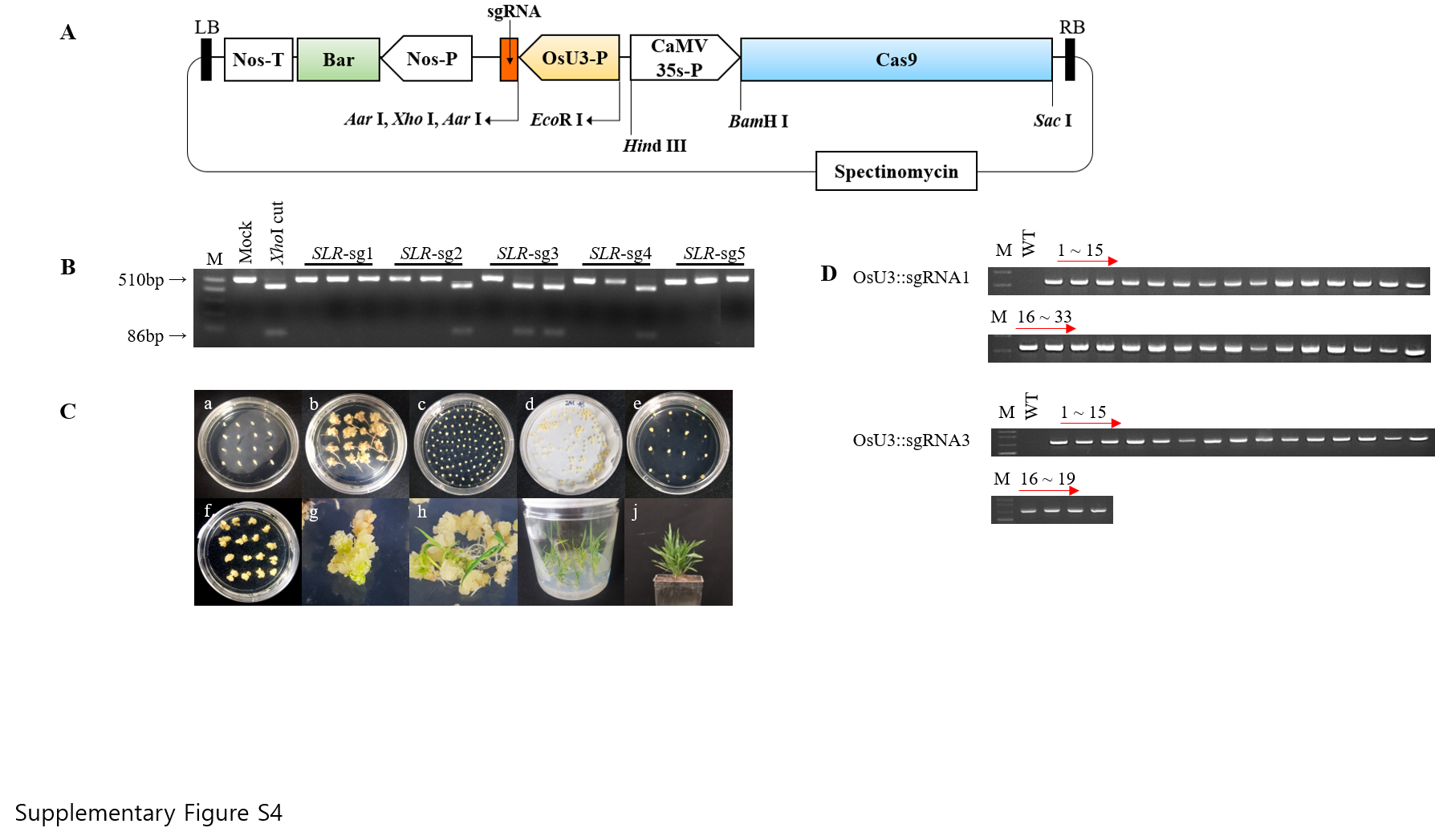

Supplement: Supplementary file 1 [file ijms-21-05492-s001.zip › Supplementary Figure S4.tif]

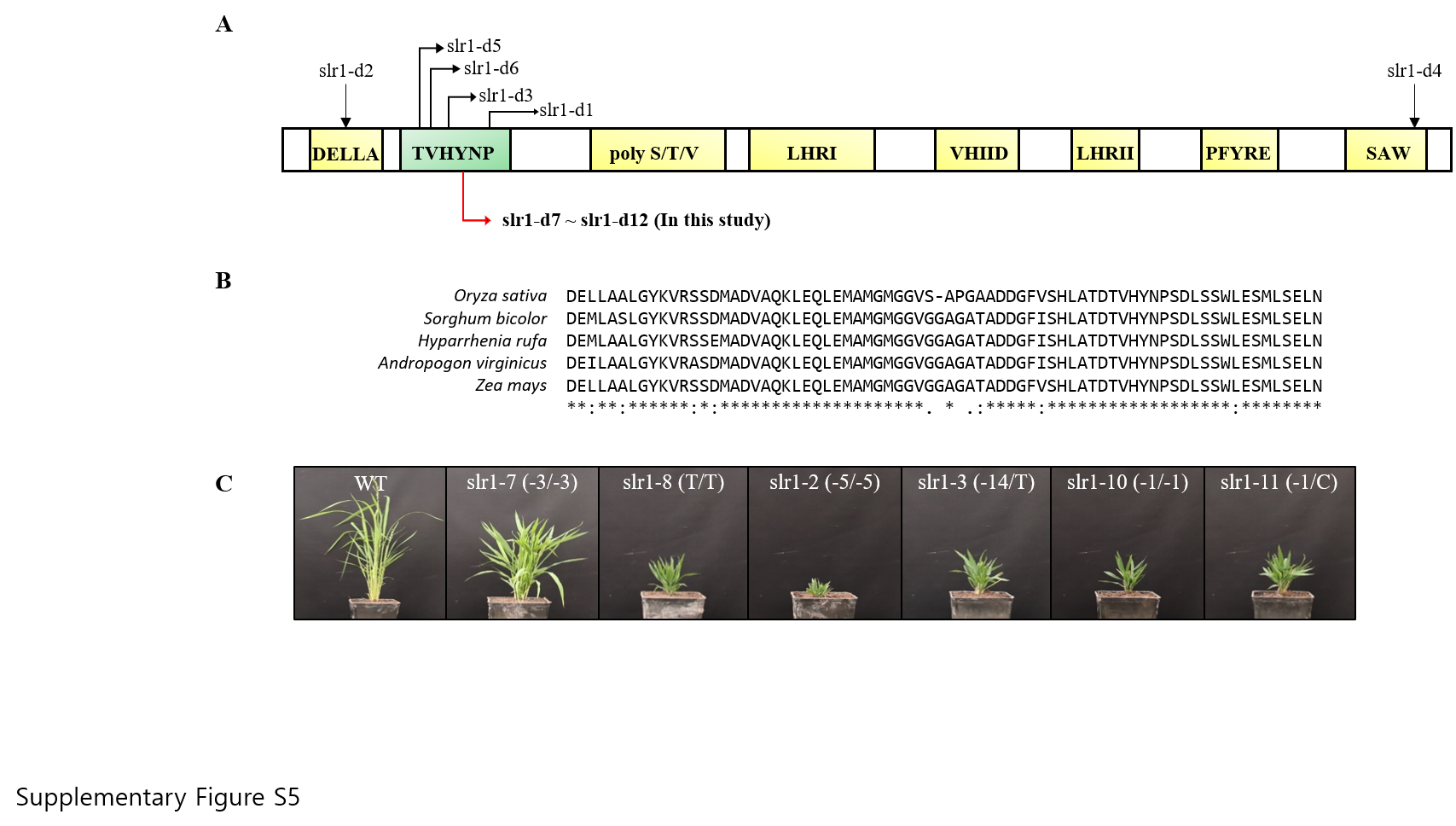

Supplement: Supplementary file 1 [file ijms-21-05492-s001.zip › Supplementary Figure S5.tif]

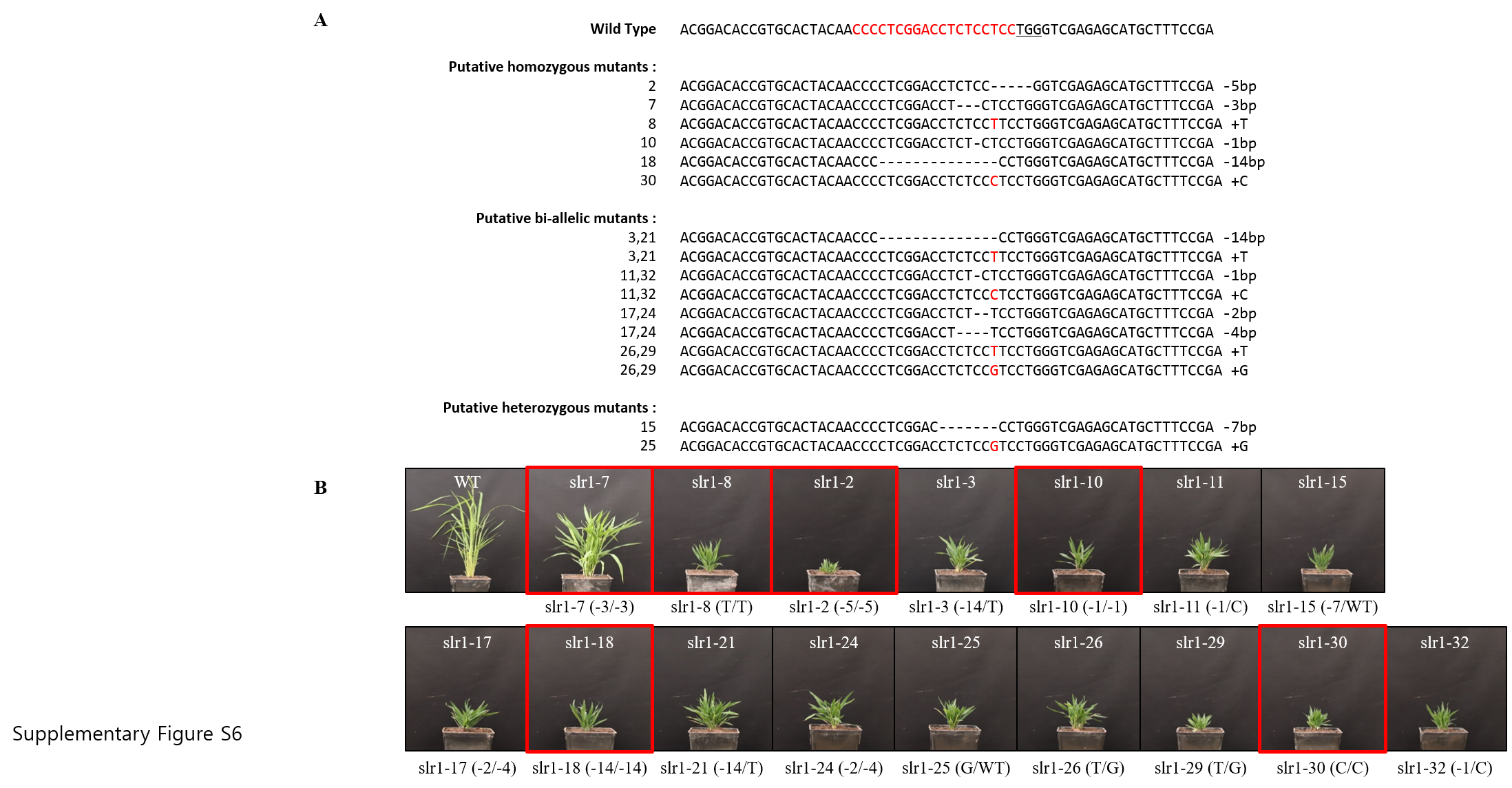

Supplement: Supplementary file 1 [file ijms-21-05492-s001.zip › Supplementary Figure S6.tif]

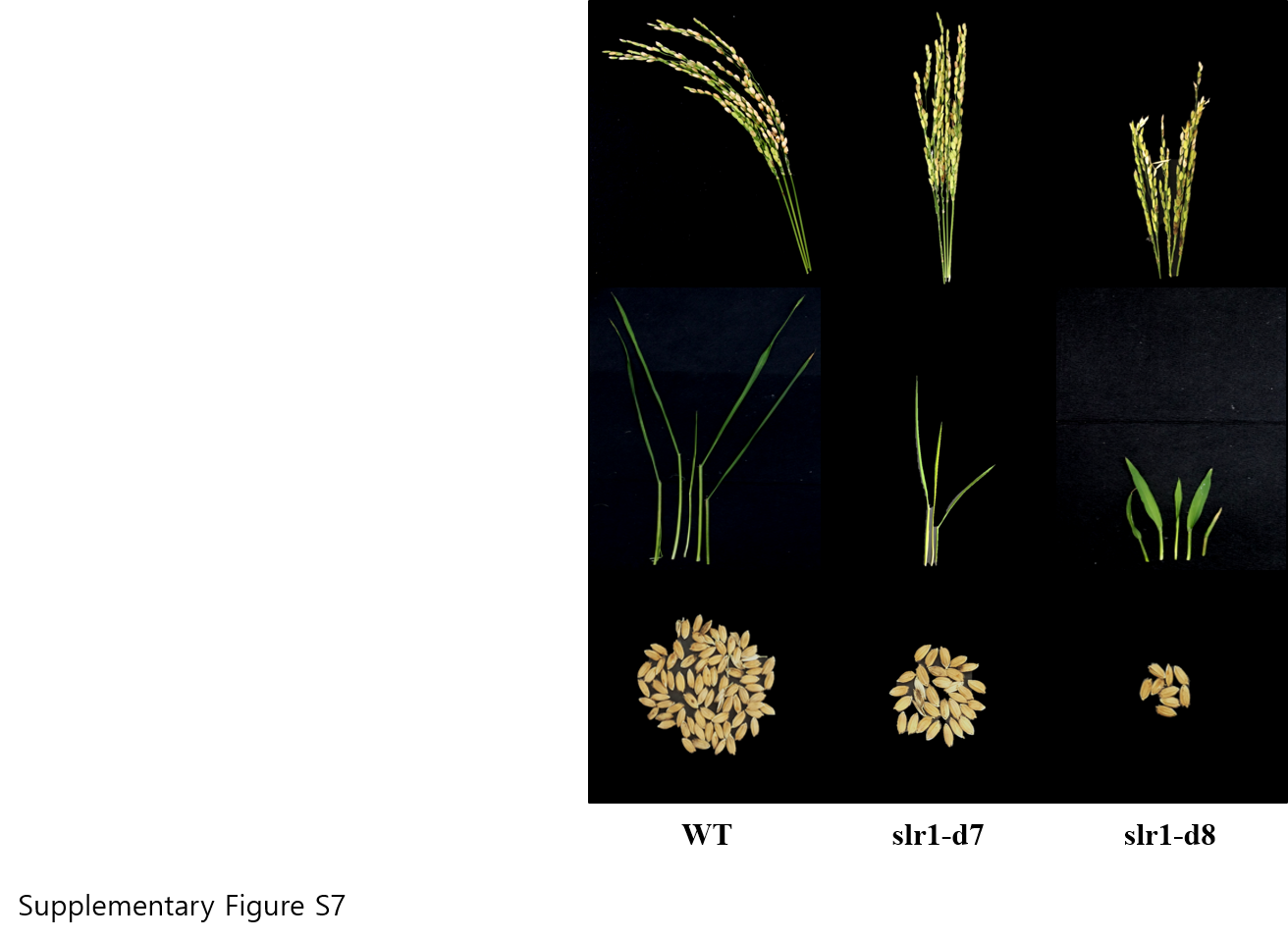

Supplement: Supplementary file 1 [file ijms-21-05492-s001.zip › Supplementary Figure S7.tif]
